# The Effectiveness of an Interprofessional Education Course in Teaching the Importance of Choosing Wisely and Resource Stewardship: A Pilot Study

**DOI:** 10.7759/cureus.14850

**Published:** 2021-05-05

**Authors:** Diane Ramsay, Yousef Bolous, Bright Huo, Emma E McDermott, Sam G Campbell

**Affiliations:** 1 Medicine, Dalhousie University, Halifax, CAN; 2 Emergency Medicine, Dalhousie University, Halifax, CAN

**Keywords:** resource stewardship, interprofessional education

## Abstract

Objectives

Rising health care costs and an increase in unnecessary testing have sparked interest in resource stewardship (RS) and subsequently the Choosing Wisely Canada (CWC) campaign. Currently, all Canadian medical schools have student representatives for CWC; however, the same is not true in other health professions. Interprofessional care learned through interprofessional education (IPE) can lead to better patient outcomes. This study assessed whether an IPE course for health profession students was effective in teaching undergraduate students both interprofessional competencies and CWC principles.

Methods

An approximately seven-hour-long, four-session course was administered to Dalhousie University health profession students (N= 30). A validated survey for IPE competencies and a general survey about CWC principles were administered to assess the course. Descriptive statistics were used to assess the general CWC views, and paired samples t-tests were employed to compare pre- and post-IPE competencies.

Results

The full survey was completed by 25 (83%) students. Of these, 52% were female, within five health disciplines, and 13 (52%) had heard of CWC prior. Overall, the students agreed that CWC was important and relevant to their profession. They also reported significant improvements in multiple IPE competencies, including communication, collaboration, roles and responsibilities, patient-/family-centered care, conflict management/resolution, and team function.

Conclusion

Participants in our pilot Choosing Wisely IPE course valued the importance of the CWC campaign and reported improvement in multiple IPE competencies. This adaptable, simple, and low-cost course may be an effective way to integrate RS teaching across multiple health professions.

## Introduction

The Canadian Institute for Health Information estimated that up to 30% of the tests, treatments, and procedures performed annually are potentially unnecessary [1], contributing to $264 billion in health care costs in 2019 [2]. Unnecessary care refers to care which does not improve outcomes, may pose harm to patients, and generates avoidable health care costs [3]. A clinician-led campaign called Choosing Wisely Canada (CWC) was launched in 2014 to help facilitate discussions around overuse, waste, and harms of unnecessary testing relevant to the professions of dentistry, medicine, nursing, and pharmacy [2]. CWC is a resource for practicing health care providers and includes lists of recommendations developed initially by physicians and followed by numerous national specialty associations to help guide clinical practice.

The CWC sought to foster medical student interest by establishing the Students and Trainees Advocating for Resource Stewardships (STARS) campaign in 2015 among Canadian medical schools [4]. Since then, the STARS campaign has grown to include medical students from all 17 medical schools across Canada. These students continue to advocate for local curricular changes in order to bring about local awareness of CWC [4]. These students have also contributed to CWC by developing a list of recommendations aimed at medical students and their needs [5]. This shows that CWC and the STARS initiative is well integrated and has had successes with medical schools in Canada. While the engagement of medical students has brought about change, student involvement in other health professions is not well understood.

Interprofessional (IP) care is known to improve patient outcomes, and effective IP care is contingent on high-quality interprofessional education (IPE) [6]. CWC engagement with allied health professions students may not be as robust because initiatives such as STARS have not been implemented in other professional schools. As such, we aimed to develop an IPE course around the principles of RS targeted at an IP student group, using the CWC campaign and recommendations. Our goal was to engage other health profession students in these conversations about unnecessary testing, treatments, and procedures, and to foster values of interprofessionalism among students.

## Materials and methods

To address the need for IP student education surrounding the principles of RS, we developed an IPE course integrated within the existing IPE curriculum at Dalhousie University. These courses are open to any health profession student and are short courses (6-9 hours) to facilitate IPE for students [7]. The course was designed by Dalhousie STARS representatives, with input from other stakeholders such as national and provincial CWC representatives, students from pharmacy, and a faculty member from nursing. Once the course was developed, a submission was approved by the IPE Coordinating Committee Executive for the 2019-20 academic year, and students self-registered for the course among a list of other courses available.

Course structure and survey design

**Figure 1:**
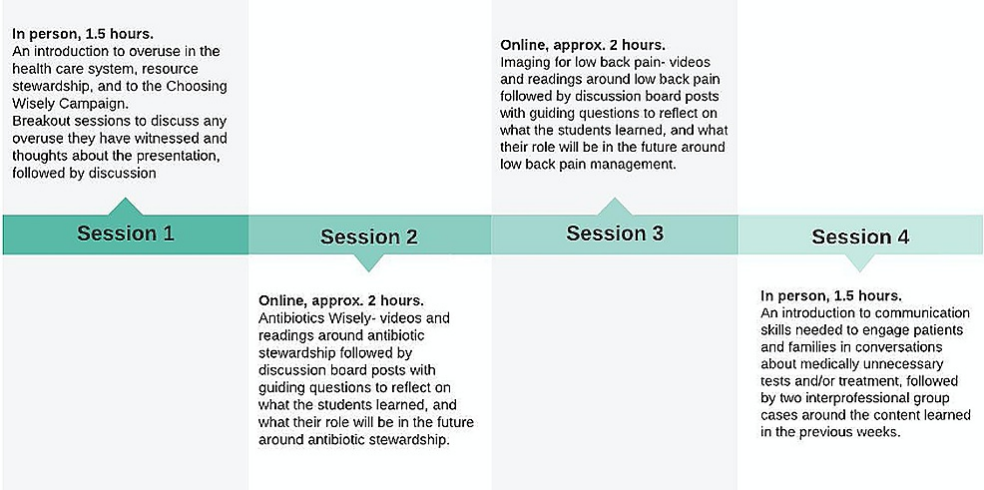
Outline of the IPE course and the sessions IPE, interprofessional education

The course consisted of four sessions, with a blended in-person and online approach. The sessions included an introduction to the CWC Campaign, Antimicrobial Stewardship (AMS), management of lower back pain, and communication skills (Figure 1). At the end of the final session, a survey was distributed to all participants to assess the IPE course. Demographic data including age, program, and year of training were collected, while the relevance and importance to participants’ professions were captured using a Likert Scale of “1, “Strongly disagree”; 2, “Disagree”; 3, “Neither agree nor disagree”; 4, “Agree”; 5, “Strongly agree”. Participants also completed the validated Interprofessional Collaborative Competency Attainment Survey (ICCAS), which assesses self-reported pre- and post-IPE competencies in communication, collaboration, roles and responsibilities, patient-/family-centered care, conflict management/resolution, and team function using a Likert Scale of 1, “Poor”; 2, “Fair”; 3, “Good”; 4, “Very good”; 5, “Excellent” [8]. As recommendations vary by discipline, students were prompted to report their perceptions surrounding the principles of CWC, which aim to promote the judicious use of health care resources.

Statistical methods and data analysis 

Data were analyzed using IBM Statistical Package for the Social Sciences (SPSS) software Version 25 (IBM Corp., Armonk, NY, United States) [9]. Only participants who completed all sections of the survey were included for analysis. Descriptive statistics (means/standard deviations and counts/percentages) were used to summarize survey responses and participant characteristics. Paired sample t-tests were employed to compare the ICCAS results before and after the IPE course, with the significance level set at α ≤ 0.05. As the primary purpose of this study was quality improvement of the IPE sessions using routine course assessment data, no Dalhousie Research Ethics Board approval was necessary, as determined by the Dalhousie Research Ethics Board “Guidelines for Differentiating Among Research, Program Evaluation and Quality Improvement” [10].

## Results

Of the 30 students who attended the course, 25 (83%) completed the survey. The participants were 52% female and were spread across five different health disciplines (Table 1). Of the 25 participants, only 13 (52%) had heard of Choosing Wisely prior to the course.

**Table 1:** Demographics

Gender	n	%
Male	12	48
Female	13	52
Program		
Medicine	5	32
Nuclear medicine	1	4
Nursing	8	32
Pharmacy	2	8
Social work	7	28

Choosing Wisely perceptions

All scores were measured on a Likert scale, and overall the students felt that Choosing Wisely was important (4.6 ± 0.7) and relevant to their profession (4.5 ± 1.1). They found that Choosing Wisely was important to their classmates (4.4 ± 1.0) and their curriculum (4.5 ± 1.0), and they also viewed that an IP approach to Choosing Wisely was important (4.4 ± 1.0). There was less agreement between students on the willingness of their professional schools to adopt Choosing Wisely into their curriculum (3.8 ± 1.1).

Interprofessional Collaborative Competency Attainment Survey (ICCAS) results

The majority (17/20) of questions for the ICCAS survey showed a significant improvement in collaborative competencies from before to after the IPE (Table 2). The three questions that did not show a significant improvement were: “Provide constructive feedback to IP team members”, “Include the patient/family in decision-making”, and “Actively listen to the perspectives of IP team members”.

**Table 2:** Pre- and Post-IPE ICCAS Results *p < 0.05. Significant factors are in bold. IP, interprofessional; ICCAS, Interprofessional Collaborative Competency Attainment Survey

Question	Pre-Score	Post-Score	p-Value
Promote effective communication among members of an IP team	3.5 ± 0.9	3.9 ± 0.8	0.001*
Actively listen to IP team members’ ideas and concerns	3.9 ± 1.0	4.3 ± 0.7	0.038*
Express my ideas and concerns without being judgmental	3.8 ± 0.8	4.2 ± 0.6	0.009*
Provide constructive feedback to IP team members	3.4 ± 0.8	3.6 ± 0.9	0.070
Express my ideas and concerns in a clear, concise manner	3.4 ± 0.8	3.9 ± 0.6	0.003*
Seek out IP team members to address issues	3.4 ± 0.8	3.9 ± 0.8	0.013*
Work effectively with IP team members to enhance care	3.6 ± 0.9	4.0 ± 0.7	0.024*
Learn with, from, and about IP team members to enhance care	3.7 ± 0.8	4.2 ± 0.8	0.009*
Identify and describe my abilities and contributions to the IP team	3.5 ± 0.8	4.0 ± 0.7	0.001*
Be accountable for my contributions to the IP team	3.6 ± 0.8	4.0 ± 0.7	0.009*
Understand the abilities and contributions of IP team members	3.4 ± 0.9	4.0 ± 0.8	0.005*
Recognize how others’ skills and knowledge complement and overlap with my own	3.5 ± 1.0	4.1 ± 0.7	0.003*
Use an IP team approach with the patient to assess the health situation	3.5 ± 0.9	4.0 ± 0.7	0.008*
Use an IP team approach with the patient to provide whole person care	3.6 ± 1.0	4.2 ± 0.6	0.001*
Include the patient/family in decision-making	3.9 ± 0.9	4.2 ± 0.8	0.129
Actively listen to the perspectives of IP team members	3.9 ± 1.0	4.2 ± 0.7	0.059
Take into account the ideas of IP team members	3.8 ± 0.8	4.4 ± 0.7	0.001*
Address team conflict in a respectful manner	3.6 ± 0.8	4.1 ± 0.7	0.008*
Develop an effective care plan with IP team members	3.4 ± 0.8	3.9 ± 1.0	0.002*
Negotiate responsibilities within overlapping scopes of practice	3.4 ± 0.7	4.0 ± 0.9	0.001*

## Discussion

The results of this survey demonstrate that a seven-hour IP mini-course yielded a significant improvement in multiple IPE competencies, as measured via the ICCAS survey, in health profession students at a Canadian University. Although three questions in the ICCAS did not show a significant improvement, our survey showed significant findings regarding the CWC principles were seen as important by the students enrolled in the IPE, and the only item which scored below 4 (4 = agree) was student perception of the willingness of their professional schools to adopt CWC principles into their curriculum. The pre- and post-IPE survey showed significant improvements in all domains of the ICCAS (domains are communication, collaboration, roles and responsibilities, patient-/family-centered care, conflict management/resolution, and team function).

Previous studies have shown that medical students benefit from learning about high-value care (HVC), resource stewardship (RS), and patient-centered care by improving their knowledge base and their confidence in discussing the evidence with patients [11,12]. Interestingly, this holds true for residents and practicing physicians [13]. There is one other Canadian university with a CWC IPE course, the University of Saskatchewan [14]; however, to our knowledge, this is the first review of such a course. The inclusion of an IPE course in undergraduate health professions curricula may fill an unmet need for education surrounding RS in order to address inappropriate testing and treatment in health care. Additionally, studies have shown medical students have expressed interest to have more education in the field of HVC and RS, both integrated into their curriculum and clerkship experience [15]. There is a lack of literature surrounding teaching RS and CWC principles to health professions outside of medicine, further supporting the utility of an IPE course, such as the one described in this study. Emerging evidence has shown that IP care can help to improve patient outcomes [6] and that early exposure to RS may lend toward improving clinical decision-making skills and developing good clinical habits [16]. As practice habits are formed early, training students’ HVC habits can improve future RS practices [16].

This course appears to have been effective in teaching both the CWC principles as well as fostering important IP skills such as communication and collaboration in a short amount of time. About half of the students had not had exposure to the CWC campaign prior to the IPE course; however, after the course, most respondents agreed that CWC was important and relevant to their profession. Additionally, the majority (17/20) of questions from the ICCAS showed an improvement in IP competencies from pre-IPE to post-IPE, demonstrating that key IP lessons were learned as well. The three questions that did not demonstrate a significant improvement were “Provide constructive feedback to IP team members”, “Include the patient/family in decision-making”, and “Actively listen to the perspectives of IP team members”. The content of the IPE did not include any chances for the students to provide feedback to their peers nor any chances to think about the patient’s family in decision-making. Future iterations of this IPE course will be re-designed to address these gaps. Our findings suggest that teaching communication skills for conversations surrounding RS may be improved by integrating simulated patients to build these skills into an IPE course in order to foster these conversations.

As this study is based on self-report, it does not formally assess whether or not students internalized the concepts presented. Although the survey yielded a high response rate, the small sample size limits the number of comparisons and conclusions that can be drawn from this survey. There is also a potential for selection bias, as students who enrolled for this course may have had pre-existing inclinations toward the CWC campaign, although this may have been mitigated, as half of the students had not had exposure prior to the course. The feedback will be utilized to develop a more comprehensive course, with an emphasis on enhancing the relevance to a wider breadth of health professions and integrating patient/family-centered care into decision-making. These changes may improve the overall quality of the course and address the gaps in this course, which were identified by the ICCAS. We hope that the interest generated with this course will increase awareness within the IP community and enhance enrollment in future courses. Future research should be conducted with larger course capacities in order to determine whether a student’s profession has an impact on their perceptions of the CWC Campaign. This model is easily adaptable to other healthcare institutions. Moreover, other schools seeking to implement similar initiatives stand to benefit from this model as it serves to promote CWC principles and IPE competencies.

## Conclusions

Participants in our pilot Choosing Wisely IPE course valued the CWC Campaign and reported improvements in multiple IPE competencies, including communication, collaboration, roles and responsibilities, patient-/family-centered care, conflict management/resolution, and team function. This adaptable, simple, and low-cost course may be an effective way to integrate RS teaching across multiple health professions, exposing students to these principles early on as future stewards of an overburdened health care system.
